# Systems Biology Applied to the Study of Papaya Fruit Ripening: The Influence of Ethylene on Pulp Softening

**DOI:** 10.3390/cells10092339

**Published:** 2021-09-07

**Authors:** Caroline Giacomelli Soares, Samira Bernardino Ramos do Prado, Sónia C. S. Andrade, João Paulo Fabi

**Affiliations:** 1 Department of Food Science and Experimental Nutrition, School of Pharmaceutical Sciences, University of São Paulo, São Paulo 05508-000, Brazil; cagias@usp.br (C.G.S.); samira.prado@oru.se (S.B.R.d.P.); 2 Food Research Center (FoRC), CEPID-FAPESP (Research, Innovation and Dissemination Centers, São Paulo Research Foundation), São Paulo 05508-080, Brazil; 3 Departamento de Genética e Biologia Evolutiva, Instituto de Biociências, Universidade São Paulo, São Paulo 05508-060, Brazil; soniacsandrade@ib.usp.br; 4 Food and Nutrition Research Center (NAPAN), University of São Paulo, São Paulo 05508-060, Brazil

**Keywords:** systems biology, papaya pulp softening, ethylene, post-harvest ripening-related modifications, pectinases, cell wall, climacteric fruit, non-climacteric fruit

## Abstract

Papaya is a fleshy fruit that undergoes fast ethylene-induced modifications. The fruit becomes edible, but the fast pulp softening is the main factor that limits the post-harvest period. Papaya fast pulp softening occurs due to cell wall disassembling coordinated by ethylene triggering that massively expresses pectinases. In this work, RNA-seq analysis of ethylene-treated and non-treated papayas enabled a wide transcriptome overview that indicated the role of ethylene during ripening at the gene expression level. Several families of transcription factors (AP2/ERF, NAC, and MADS-box) were differentially expressed. ACO, ACS, and SAM-Mtase genes were upregulated, indicating a high rate of ethylene biosynthesis after ethylene treatment. The correlation among gene expression and physiological data demonstrated ethylene treatment can indeed simulate ripening, and regulation of changes in fruit color, aroma, and flavor could be attributed to the coordinated expression of several related genes. Especially about pulp firmness, the identification of 157 expressed genes related to cell wall metabolism demonstrated that pulp softening is accomplished by a coordinated action of several different cell wall-related enzymes. The mechanism is different from other commercially important fruits, such as strawberry, tomato, kiwifruit, and apple. The observed behavior of this new transcriptomic data confirms ethylene triggering is the main event that elicits fast pulp softening in papayas.

## 1. Introduction

In addition to fruit ripening incrementing sensory and nutritional quality, it also increases fruit susceptibility to physical damage as the pulp and skin soften [1]. The ripening process consists of a series of biochemical transformations favoring the phenotype to attract consumer animals that promote seed dispersion, as well as reaching flavor, odor, texture, color, and nutritional quality suitable for consumption [2,3]. Fleshy fruits are divided into two categories based on how the ripen: climacteric and non-climacteric. It depends on the presence (climacteric) or absence (non-climacteric) of rise in respiration and ethylene production [2]. Ethylene coordinates the ripening process but is also known to regulate gene expression on several stages of fruit development [4,5,6]. The transduction pathway initiates when ethylene binds to a specific receptor, that will initiate the signaling cascade by releasing CTR1 and EIN2 binding, activating several transcription factors (EIN3/EIL1 and ERFs). These transcription factors regulate genes underlying ripening-related traits, such as pulp firmness [2,3,4,5,6]. Ethylene exogenous treatment rapidly increases the transcription of all these genes regulated by this hormone [5,6,7,8]. In this way, an effective methodology to investigate the expression profile during ripening is transcriptomics, which identifies the complete set of a sample’s mRNA content. Deeper knowledge about the genetic control of ripening is limited to a few fruits, such as the model fruit tomato, but several studies have already been conducted with kiwifruit, apple, strawberry, melon, mango, peach, and banana, among others [5,6,9,10,11,12,13,14]. Some tropical fruits of great commercial importance, such as papaya, are still restricted to a superficial knowledge on a global expression profile, with most research focused on the study of the expression of specific enzymes [1,4,15,16].

Papaya (*Carica papaya* L.) is a fleshy fruit and a plentiful source of carotene and vitamins A and C. When ripe, the fruit is usually consumed raw but also used to make jams, candies, juice, and to isolate papain and bioactive polysaccharides [17,18]. However, the market of this fruit is greatly affected by post-harvest losses due to excessive softening of the pulp, which favors microbial attack and mechanical damage. Because papaya is a climacteric fruit, the pulp and peel softening occur relatively quickly, and management techniques are limited in terms of preventing and minimizing damage [8]. Elucidating the structure, functions, and gene expression regulation of the plant cell wall during fruit ripening is essential as commercial interests in postponing the shelf-life storage are crucial to maintaining the sensorial fruit quality [7]. Although papaya has most of its genome sequenced, many studies focused solely on changes in the cell wall that culminate in pulp softening [19,20,21,22]. The transcriptome of papayas treated with ethylene and its inhibitor (1-methylcyclopropene) revealed the main set of genes affected by the lack of ethylene triggering, but several ripening-representative physiological parameters were not analyzed [23]. Therefore, the present study aimed to establish a general panorama of gene expression in a ripening stimulation concept through ethylene-treated papayas based on RNA-seq technology. We integrated our RNA-seq data with four other studies aiming to develop new perspectives on the complex physiological phenomenon of papaya ripening at a transcriptional level, different from some climacteric and non-climacteric fruit models (e.g., tomatoes and strawberries, respectively).

## 2. Materials and Methods

### 2.1. Plant Materials and Experimental Design

Papayas (*Carica papaya* L. cv. “Golden”) at the pre-climacteric stage were acquired from a commercial producer in the municipality of Linhares/ES, Brazil, (19°21′33.9″ S 40°08′15.6″ W) between one and two days after harvest, still with up to one-quarter of yellow peel (about 150 days after anthesis) and were harvested in three consecutive harvesting times (August, September, October 2017). Right after the fruit arrived in the laboratory and following sanitization with chlorine (100 ppm), some were immediately characterized according to Fabi et al. [24] and frozen to compose the 0-h control group. Remaining fruits were separated into two groups: treated and control. Control groups were left to ripen with the parameters described in Fabi et al. [24], and fruit samples were taken after 12 h and 24 h. Treatment with ethylene at 0 h was done according to Fabi et al. [24] by exposing randomly selected fruit to a concentration of 100 ppm (100 μLL^−1^) of ethylene, kept in constant flux for 17 min for gas saturation and 12 h more in a closed system. After 12 h, fruits were removed from the ethylene chamber and exhaustively air-vented for 1 h. Ethylene-treated fruits were frozen (12 h group), and the other set of fruit was left to ripen as the control group for 12 more hours, totaling 24 h after fruits reached the laboratory (group 24 h treated). In all five groups (0 h, 12 h control, 24 h control, 12 h ethylene, and 24 h ethylene), papayas were characterized for ripening parameters and peeled; their seeds were removed, and the pulp was cut into cubes, frozen in liquid nitrogen, and stored at −80 °C (Figure S1). The experiment used three sets of harvested fruit collected at three different times of the year (biological triplicate), and each group was composed of at least five fruits (which were pooled before storage at −80 °C).

### 2.2. Analysis of the Ripening Parameters

The ethylene produced in the head space of a chamber containing one papaya was analyzed through flame ionization gas chromatography (GC-FID) following the methodology described in Fabi et al. [24]. In addition to visual inspection, a colorimeter (CR 410, Konica Minolta, Tokyo, Japan) was used to measure the color of the fruit peel. Following the CIE system, a, b, and L parameters were taken from six spots and then transformed in the hue angle, which was high if the peel was green and low if it was yellow [24]. The peel resistance was measured using a penetration probe of 9 mm diameter at three different spots on the fruit, representing the whole fruit’s thickness. Then, fruits were cut longitudinally, and pulp resistance was measured using a penetration probe of 6 mm diameter at six different spots [24].

### 2.3. Total RNA Extraction

The total RNA of the fruit was isolated using the Concert™ Plant RNA Reagent (Invitrogen^®^, Carlsbad, CA, USA) and purified by treatment with the DNA-free™ kit (Invitrogen^®^, Carlsbad, CA, USA), following the protocol described by the manufacturer. Nucleic acids were quantified spectrophotometrically using the Implen^®^ (Westlake Village, CA, USA) N50 spectrophotometer. Quality was assessed by the absorbance readings at 260 nm/280 nm and 260 nm/230 nm and evaluated through 1% agarose gel electrophoresis. The RNA samples were subjected to second quantity, purity, and quality analyses using the Agilent 2100 Bioanalyzer (Agilent Technologies ©, Santa Clara, CA, USA).

### 2.4. Illumina Sequencing (RNA-Seq)

The cDNA libraries were sequenced at the Center of Functional Genomics, ESALQ-USP (Piracicaba/Brazil). A total of 15 cDNA libraries were prepared using the Illumina TruSeq Stranded mRNA LT Sample Prep Protocol and paired-end sequenced using HiSeq SBS Kit v4 on the HiSeq2500 system (Illumina, San Diego, CA, USA). These libraries were deposited under access number GSE128577 at GenBank. The raw RNA-seq data was initially filtered by SeqyClean version 1.10.09 and checked for quality through FastQC software v. 0.11.8. The alignment of the reads against the papaya reference genome, available at NCBI (Papaya1.0, GCF_000150535.2), was performed by the software STAR v. 2.6.0a to obtain the uniquely aligned reads [19,25,26,27]. The BlastX tool was used to align the extracted transcript sequences to Viridiplantae (txid33090) non-redundant (nr) database.

### 2.5. Differential Expression Analysis

For the statistical analysis of the differentially expressed genes, the DESeq2 package v.1.20.1 from R software v.4.0.3, specific for differential expression analysis, was used [28,29]. The comparisons were completed between all treatments, thus developing a temporal overview of ripening, and verifying the effect of the treatment. Genes with adjusted *p*-values (*p*-adj) ≤ 0.05 were considered differentially expressed. Ethylene-related and cell wall-related genes with *p*-values ≤ 0.05 were considered differentially expressed.

### 2.6. Enrichment Analysis

All genes were annotated and mapped to referent Gene Ontology (GO) terms using the Blast2GO tool v. 5.2.5 [30]. The identified differentially expressed genes with log2FC ≥ |1.5| and adjusted *p*-value ≤ 0.05 were submitted to GO enrichment analysis. Fisher’s exact test was performed in two-tailed mode to detect both over and underrepresented GO terms with a threshold of FDR ≤ 0.005 for the categories biological process, molecular function, and cellular components.

### 2.7. Co-Expression Analyses

We conducted a co-expression analysis that assigned genes to modules that were correlated to physiological data (ethylene, pulp firmness measurements and peel color) and assigned aleatorily different colors for each module. Further construction of a weighted gene correlation network was followed by using the weighted correlation network analysis (WGCNA) v. 1.69 R package [31]. The visualization of the correlation network was built using Cytoscape software v. 3.7.2 [32].

### 2.8. Differential Expression Analysis through Real-Time PCR

The experiments were rigorously performed according to the previous description [22] and using the RNA-seq samples and the ripening samples according to Prado et al. [22]. The genes used were as follows, with primers sequences, GenBank access, and efficiency curves listed in Tables S1–S3: Gene9059–PG1; gene9058–PG2; gene5336–PG3; gene9513–PL1; gene171–PL2; gene14164–AGAL1; gene1360–AGAL3; gene7019–BGAL1; gene2838–BGAL3; gene13517–PME1; gene3087–PME2; and gene15057–PME3.

### 2.9. Statistics

Experimental results are expressed as the mean ± standard deviation (SD) obtained from all fruit of each one of the five groups from the three biological replicates, with *p* < 0.05 representing statistical significance. Data were analyzed using GraphPad Prism 6.0 software (GraphPad Software, San Diego, CA, USA). One-way analysis of variance (ANOVA) with Tukey’s test (to assess differences among all groups) was used as a post hoc test. Differential expression analysis followed the empirical Bayes approach to assign moderated estimation of dispersion and fold change values of pairwise comparison between all samples, while co-expression assay was based on a Pearson correlation [29,31]. Both analyses were performed considering the whole transcriptome of the samples: posteriorly selected genes of interest based on a Blast similarity of *p*-value ≤ 10^−7^. Blast2GO software was used to run an enrichment analysis through Fisher’s exact test with a cutoff of *p*-value ≤ 0.05 [30]. Co-expression correlations greater than 0.05 were considered significant. The network visualization on Cytoscape was designed by an algorithm called Edge-weighted Spring-Embedded Layout, which treats the weight of correlations as an inverse physical force, resulting in smaller edge lengths to higher correlations between nodes [32].

## 3. Results and Discussion

### 3.1. Ethylene Treatment Induced Papaya Ripening

**Figure 1:**
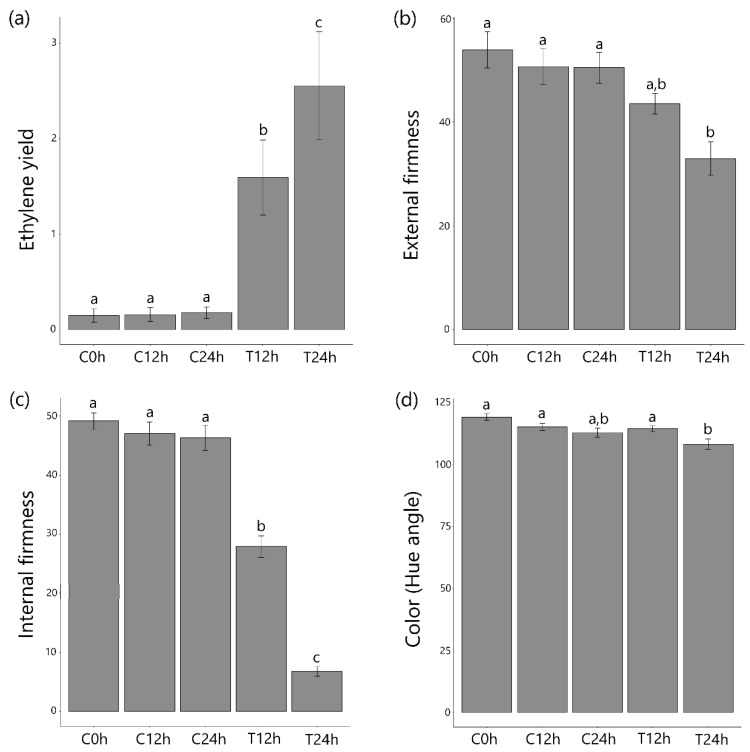
Ripening parameters measured for papaya treatment in the biological triplicate. Ripening parameters: (**a**) Ethylene yield (µL, Kg^−1^, h^−1^), (**b**) external firmness (N), (**c**) internal firmness (N), and (**d**) pulp color (hue angle). Six papayas were used for each time point (0 h, 12 h, and 24 h) for each biological replicate. Shown on *y*-axis the average of three biological replicates ± Standard Error. Samples are identified as C (control) or T (treated) followed by the time point. Different letters (a,b,c) represent significant differences in values (Fisher test at *p* < 0.05).

Papayas are climacteric, fleshy fruit characterized by fast pulp softening in the post-harvest period [24]. As expected, ethylene treatment triggered the self-production of endogenous ethylene in the treated group after 12 h and 24 h, while in the control group, similar levels were maintained over time (Figure 1; Figure S2). Texture measures from both whole fruit (external firmness) and fruit pulp (internal firmness) exhibited decay in treated fruits, while no changes were seen in the control group. Significant skin color changes were only detected in one replicate (B). Usually, papayas completely change their peel color after greater ripening time [3]; therefore, due to the short period in which the samplings were completed, significant differences in the papayas’ peel color were not expected. In general, the results obtained were consistent with expectations after treatment with ethylene and simulating the post-harvest phase. Sampling B showed minimal differences in 12 h sample maybe due to variation in growing and harvesting conditions that resulted in an earlier stage of ripening even being at ~150 days after anthesis. Nevertheless, it is still possible to notice the initiation of the ripening process due to increase in ethylene yield and decrease of pulp and peel firmness after 12 h of ethylene post-treatment (sample T24h). These results allow for a description of the maturity profile for the sample, which consisted of fruit of the same origin and at the same time of the year. Furthermore, the results enabled the collection of plant materials corresponding to the three biological replicates for RNA-seq analysis. The values obtained for all the analyzed parameters indicated the homogeneity of all samples, a fact that confirms the similarity of the stage of ripening of the fruit when harvested and, mainly, unifies the ripening process [24,33].

### 3.2. Changes to Transcriptome Profile during Ripening

Approximately 167.9 million fragments were uniquely mapped against the reference genome (GCF_000150535.2, disposed of 370.5 million base pairs with a size of 372 mega-bases), corresponding to about 86% of the fragments that were kept after the filtering process, while just over 13% were not mapped [19]. Only 1736 of 23,332 genes on the reference genome had no fragment aligned. A BLASTx against the non-redundant data set from NCBI identified 19,316 genes, a second search against the *Arabidopsis thaliana* genome resulted in 18,378 homologous genes. After a manual screening, only 1355 genes remained unidentified. A total 12,150 genes were assigned to 5349 GO terms within the categories of Biological Process (3020), Molecular Function (1695), and Cellular Component (634). Functional pathways with the highest number of genes refer to cellular and metabolic processes, responses to chemical signals and stress, system development in general, binding, catalytic activity, transcription regulation, and intracellular components and organelles (Figure S3).

**Figure 2:**
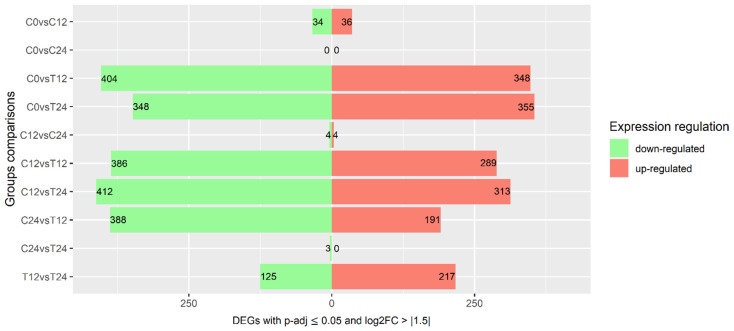
Differentially expressed genes. Total number of differentially expressed genes with *p*-adj ≤ 0.05 and log2FC > |1.5| between pairwise comparisons. Green bars represent downregulated genes, while red represent upregulated genes. Samples are identified as C (control) or T (treated) followed by the time point.

Pairwise differential expression analysis between all control and treated samples identified 8936 and 8726 significantly up- and downregulated genes, respectively. In a refined perspective, the number of genes with expression of at least 50% of variation in log2FC (log2FC > |1.5|) is represented in Figure 2 (1753 upregulated and 2104 downregulated genes) (Supplementary File 2). Comparisons between control and treated samples revealed a higher number of DEG, while differences between control samples remained minimal, suggesting that exogenous ethylene enhanced gene transcription.

Differentially expressed genes between control groups are related to anatomical structure development and RNA metabolic processes. Considering the comparisons between control and treated samples, upregulated genes had a positive regulation for programmed cell death, camalexin biosynthetic process (pathogen defense), abscisic acid metabolic process, plant-type cell wall organization with the activity of xyloglucan: xyloglucosyl transferase and UDP-glucosyltransferase, root development, and leaf senescence. In contrast, repressed pathways of upregulated genes were generally involved in translation. Ethylene-treated samples’ expression underwent downregulation in genes enriched to negative regulation of cell growth, inflorescence morphogenesis, and external stimulus, while translation and intracellular protein transport decreased.

### 3.3. Correlation among Expression and Phenotypic Data

**Figure 3:**
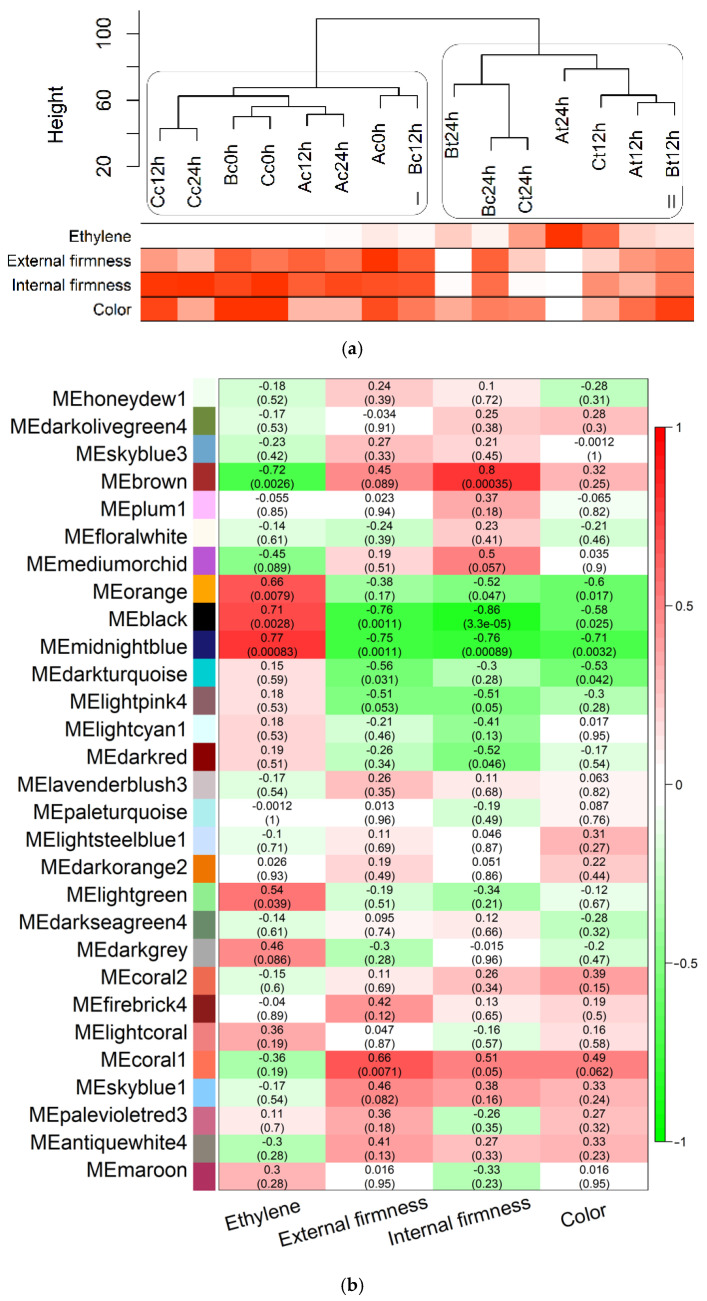
Sample dendrogram of transcriptomic data, phenotypic heatmap, and module-phenotype correlation: (**a**) Gene expression clustering of samples organized into two main clades (I—Control; II—Treated) and heatmap of the ripening parameter’s measures, in which white is low values and red is high values; (**b**) Heatmap of the correlation between modules and physiological measures. Colors on the left outside column represent the modules of genes with similar expression profile. Each cell informs the correlation level (number on top; positive correlation: red; negative correlation: green) and *p*-value (number on bottom, in parenthesis). Samples are identified as capital letters A, B, or C according to the three biological replicates, followed by lower case letters c (control) or t (treated) and the time point (0, 12 or 24 h). Other details are described in the Materials and Methods.

After clustering the samples through gene expression and phenotypic data, control and treated samples were separated into two main clades (I and II, Figure 3a), demonstrating that exogenous-ethylene treatment resulted in large, plain modifications in the gene expression profile. Sample C24h from sampling B was an exception probably due to the slightly increased level of ethylene yield of this sampling, which might have led to stimulate the transcription of genes similarly to ethylene-treated samples. This exception can also be found in PCA analysis of transcripts (Figure S4), which resulted in low levels of differentially expressed genes between C24h vs. T24h (Figure 2). An ethylene production rate increase is evident in the treated groups, while external and internal firmness displayed the opposite behavior. Peel color became slightly light, even though changes were minimal, probably because peel color is not uniform, and the plotted value is the mean of hue angles of several points of the fruit [24].

Modular analysis by expression profile correlation identified 29 modules with 40 (dark olive green) to 7649 (brown) members. Colors were automatically assigned to modules for representational purposes only and they do not imply any correlation between similar colors. Further correlation among modules and phenotypic parameters are represented by red if positive correlated, and green if inverse correlated in a heatmap that followed the phenotypic heatmap (Figure 3a) pattern: modules with a high correlation to ethylene yield are inversely correlated to other phenotypes (Figure 3b). In each cell, the value of the correlation between the respective module and phenotype parameter and its *p*-value is shown. Significant positive correlations (*p*-value ≤ 0.05) were detected in modules brown, orange, black, midnight blue, and light green to ethylene yield, as well as negative correlations to the other three parameters (except light green). This opposite correlation pattern suggests that, in addition to the ethylene-related genes, members of these modules are also involved in ripening processes, such as cell wall degradation and carotenoid synthesis.

Enriched pathways of genes in the black module are associated with processes of uracil catabolism and lipid and fatty acid metabolism. The activity of cellular differentiation and development and plant-type cell wall organization or biogenesis were repressed. Midnight blue members are assigned to high involvement in antioxidant activity, like vitamin E and fat-soluble vitamin biosynthetic process. Light green genes positively participate in the primary metabolic process and are negative in transcription. Orange and brown modules had no significantly enriched pathways. Genes and functional pathways of the primary metabolism with positive regulation, such as fatty acid and lipid metabolism, suggest increased activity related to the ripening process with a consequent increase in energy consumption of the fruit [34]. Lipid content is also associated with carotenoid level, thus the negative correlation of the black module with color measurements indicates an increase in major carotenoids and a decrease of total chlorophyll in yellow-peel fruit [35]. A negative correlation between the black module and firmness, together with the repression of plant cell wall organization and biogenesis, endorses the disassembling of the plant cell wall during ripening [22].

Carotenoid-accumulation is the major factor in color changing in papaya fruit, and it acts as an antioxidant. It was demonstrated that the amount of carotenoid-accumulation is highly and positively associated with ethylene levels, while its synthesis is related to lipid, fatty acids, and pyruvate metabolism, as well as lycopene biosynthetic process [36]. In our experiment, ethylene-treated samples exhibited an enhancement of pulp color and the over expression of many carotenoid-related genes, with functional pathways that were enriched. In addition, there were two carotenoid-related genes acting as hub genes in the black module.

Aroma enhancement is significantly represented by the enrichment of alcohol dehydrogenase and linalool synthase activity, which is the main volatile compound of papaya and is directly induced by ethylene [37]. In our experiment, although papaya is popular for its sweet taste, the sucrose metabolic process was suppressed in treated samples while many functional pathways of sucrose and glucose were enriched for downregulated genes. Still, there were a few upregulated genes encoding sucrose synthase as described in Gomez et al. [38]. Despite total soluble sugar content not changing much after harvesting, sucrose synthase maintains its activity by consuming carbon from the degalactosylation of cell wall polysaccharides. Another factor that makes papaya a highly consumed fruit is its antioxidant and nutrient properties. Although the maturity of papaya leads to a decrease in flavonoids and phenolic content, our data show that related genes and functional pathways induced in the treated 12 h sample were detected [39]. We have showed that gene-related vitamins A, C, and E also had their pathways enriched and were previously related to increases during ripening [8].

### 3.4. Ethylene-Related Genes and Transcription Factors

**Figure 4:**
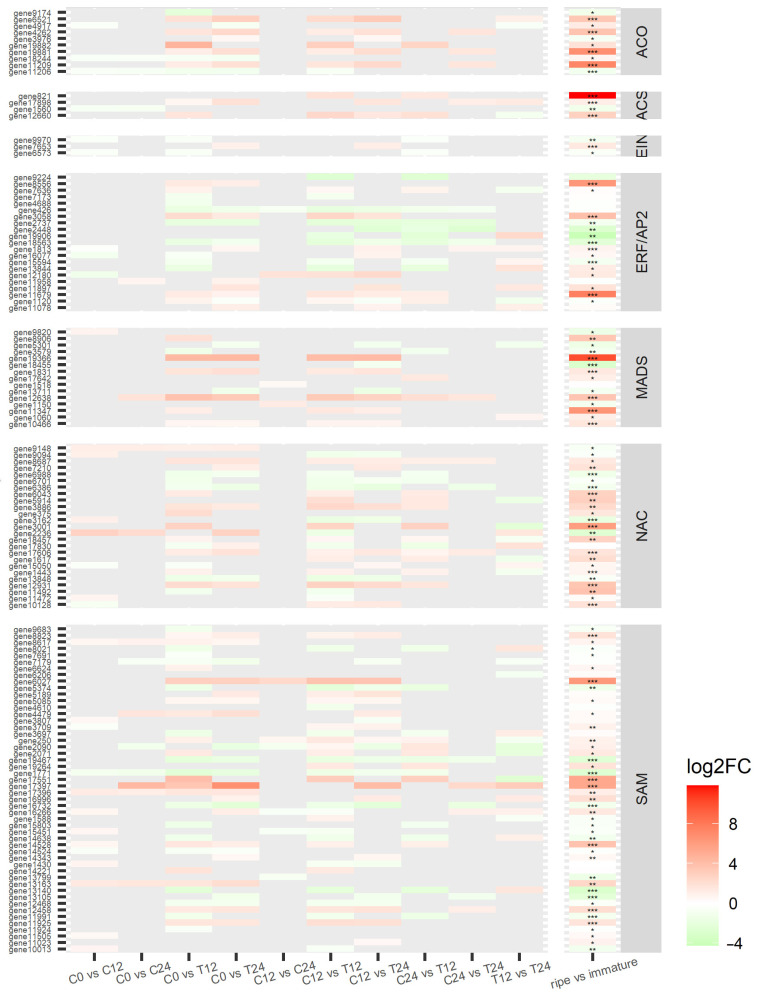
Ethylene-related genes’ expression. Colors represent the difference in expression of the latter group in relation to the first. The first set of columns is the comparisons between samples of the performed RNA-seq, and the last column refers to the differential meta-analysis. Genes are grouped by their coding enzymes: ACO, 1-aminocyclopropane-1-carboxylate oxidase; ACS, 1-aminocyclopropane-1-carboxylate synthase; EIN, ethylene-insensitive protein; ERF/AP2, ethylene-responsive transcription factor; MADS, agamous-like MADS-box protein; NAC, NAC domain-containing protein; and SAM, S-adenosyl-L-methionine-dependent methyltransferases superfamily protein. Asterisks represent *p*-adjusted values (* *p* < 0.05; ** *p* < 0.01; *** *p* < 0.001); no asterisk means *p*-adj > 0.05. Samples are identified as C (control) or T (treated) followed by the time point.

Transcription factors related to ethylene response were abundant and presented strongly different levels of expression (all differentially expressed transcription factors are depicted in Figure 4 and Table S4). ACO, ACS, and SAM-Mtase were predominately upregulated, indicating a high rate of ethylene biosynthesis. EIN is a mediator between ethylene synthesis and signaling, acting as a ‘gateway’ when CTR1, a negative regulator, is reduced by the presence of ethylene. However, there were few EIN genes differentially expressed, and they were mostly repressed. Thus, ethylene signaling operates through other routes independent of CTR1 or EIN [40]. AP2/ERF-encoding genes are involved in abiotic stress response and are particularly active during development and ripening processes [41]. From AP2/ERF family, almost all RAPs and half of the ERFs were repressed while other ERFs and AP2 were upregulated. The NAC family is one of the largest families of plant transcription factors whose genes regulate several processes of development and maintenance of the system from embryogenesis until leaf senescence [42]. NAC genes related to the regulation of the development of secondary cell wall structures and several stress responses and auxin signaling were upregulated, while the ones involved in the response to cold and pathogens were repressed [42,43]. The MADS-box family is also a large TFs family involved in the regulation of many routes and had its gene expression mostly increased. In contrast, the positive feedback to the autocatalytic cycle of ethylene in papayas is not regulated by the MADS-box family, but by a NAC gene [44]. As these TFs families are too vast and the specific function of each gene is still not completely elucidated, the assumption is that MADS-box DEGs are related to activities other than ethylene signaling regulation. For comparison, all DEGs were also identified in the differential analysis of the explored papaya datasets. Although not all DEGs in the RNA-seq conducted in this study were significantly differentially expressed in the meta-analysis, an overall pattern was apparent in both analyses (Figure 4). The similarity in the profile expression of the ethylene-related genes supports the results obtained through transcriptomics.

### 3.5. Transcriptomic Analysis Reveals a Diverse Expression of Papaya Cell-Wall-Related Genes

**Figure 5:**
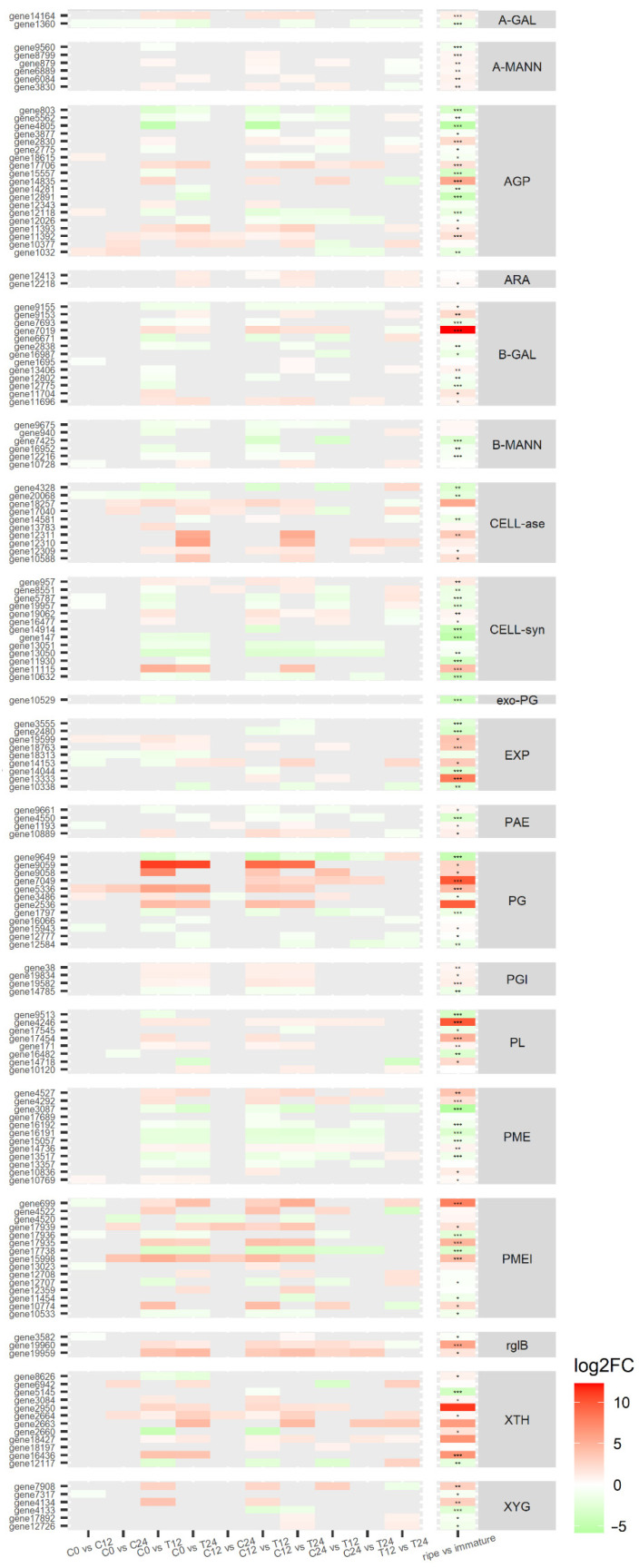
Heatmap of cell wall genes differentially expressed. Heatmaps of the expression levels of the gene coding for each enzyme related to the cell wall in both authorial transcriptomics and meta-analysis of mined datasets (last column). Colors represent the difference in expression in the latter group in relation to the first. The enzymes are represented as follows: A-GAL, alpha-galactosidase; -MANN, alpha-mannosidase; AGP, arabinogalactan; ARA, alpha-L-arabinofuranosidase; B-GAL, beta-galactosidase; B-MANN, beta-mannanase; CELL-ase, cellulase; CELL-syn, cellulose synthase; exo-PG, exo-polygalacturonase; EXP, expansin; PAE, pectin acetylesterase; PG, polygalacturonase; PL, pectate lyase; PME, pectinesterase; PMEI, pectinesterase inhibitor; rgB, rhamnogalacturonate lyase B; XTH, xyloglucan endotransglucosylase/hydrolase; and XYG, xyloglucan galactosyltransferase. Asterisks in last column represent p-adjusted values (* *p* < 0.05; ** *p* < 0.01; *** *p* < 0.001); no asterisk means *p*-adj > 0.05. Samples are identified as C (control) or T (treated) followed by the time point.

Papaya pulp transcriptome revealed 157 differentially expressed genes (*p*-value ≤ 0.05) related to cell wall metabolism according to the blasted transcripts, including some up and downregulated ones, as well as some genes known to be ethylene-induced (all differentially expressed genes related to cell wall are depicted in Figure 5 and Table S5). In general, genes related to cell wall disassembly did not change their expression in the first 12 h or 24 h of the ripening process in the control fruit. This finding corroborates the not observed changes in ethylene production and pulp texture (Figure 1), with no apparent plant cell wall alteration in the first and second days after harvesting [22]. In ethylene-treated fruit (both 12 h and 24 h groups), a considerable set of genes from PG, rhamnogalacturonate lyases, galactanases, and XTH were upregulated, while most of the PL and PME genes did not change their expression or were downregulated. A similar behavior was observed in the meta-analysis of differentially expressed genes in all integrated transcriptomes (last column of Figure 5).

Other relevant results were the upregulation of PME inhibitor genes, while the PG inhibitor was downregulated. The upregulation of rhamnogalacturonate lyase genes, enzymes responsible for hydrolyzing RG-I portions of pectin through the β-elimination mechanism, was particularly of interest. Despite being the first time, these transcripts were found in papaya pulp, they were previously identified in tomatoes and potatoes [45].

**Figure 6:**
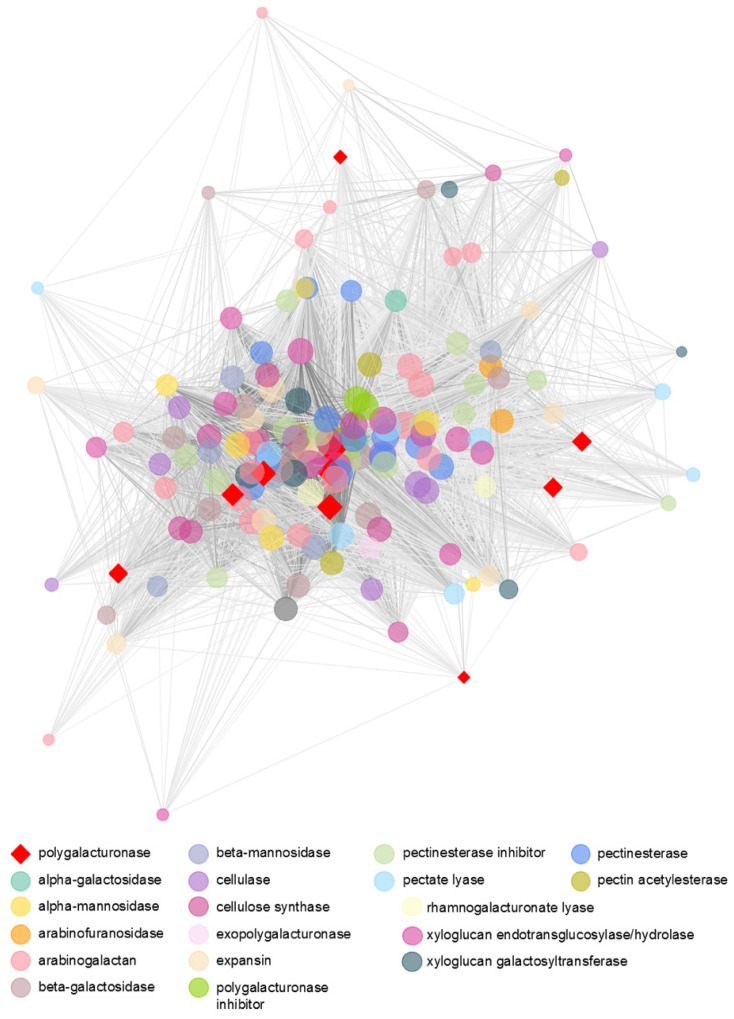
Co-expression correlation network of cell wall genes. Node sizes represent their connectivity degree. Edges vary in length and width, so the smaller and thicker an edge, the higher the correlation between the nodes.

Network files generated by WGCNA were visualized through Cytoscape with the application of the Edge-weighted Spring-Embedded Layout algorithm (Figure 6) [31,32]. Node sizes are set by their connectivity degree, representing the total number of correlations in which they participate; their color indicates the enzyme to which the node (gene) is related. Edge lengths are inversely proportional to weight values of correlations, while their width is directly proportional: the smaller and thicker an edge, the higher the correlation between the nodes.

Overall, 8294 interactions with a weight ≥0.05 between genes related to cell-wall-modifying enzymes were detected (Figure 6). The PGs, highlighted in red in the shape of a diamond, interact with almost all genes in the network except for some arabinogalactan-protein, expansins, and PLs, among others. It was observed 1176 interactions between PGs and the other genes, as well as 44 interactions between each PGs. The full table that depicts the PGs interactions with their parameter values are in Supplementary File 3. The centrality of the deeply connected PG are apparent in the center of the network, while other PGs are distributed around, indicating a heavy influence on the behavior of other members. The only exo-PG exhibits an interesting template: it is intensely correlated to the nodes in the center of the network but not to the peripheral ones. PMEs are highly connected to the network, indicating no correlation with only four genes of the 157 genes related to cell-wall enzymes, and PME inhibitors also followed the same pattern of correlation. PLs, although present in a much smaller number, also interact with almost all members of the network. Galactosidases, xylanases, and cellulases are fully interconnected with all members of the network. The rhamnogalacturonate lyases had an intense correlation mainly with genes encoding xylanases, PG, PME, expansins, cellulose synthases β-galactosidases, PG and PME inhibitors, and arabinogalactans.

**Figure 7:**
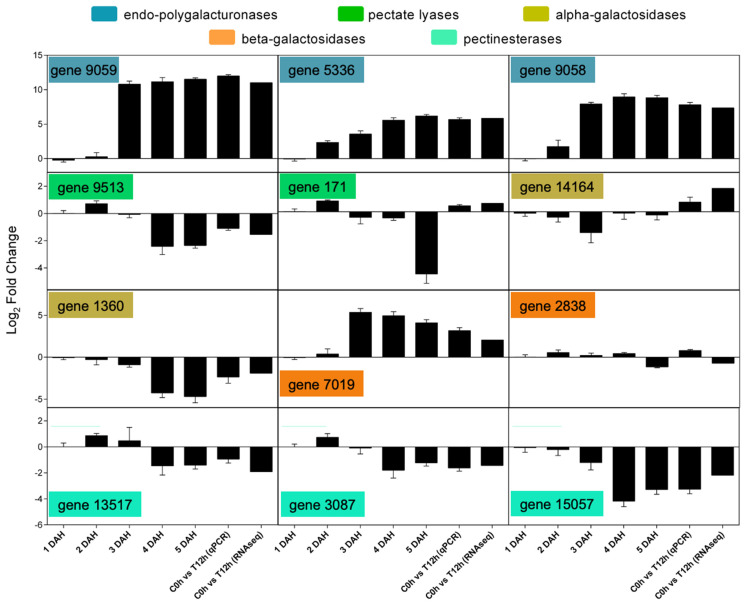
Confirmative gene expression experiment of cell-wall-related genes that were differentially expressed after ethylene-treatment of papaya fruit. The expression of differentially expressed genes between the control group (0 h) versus ethylene-treated fruit (12 h) was confirmed in the same sample (C0h X T12h) and in a ripening curve previously published with quantitative real-time PCR performed according to Prado et al. [22]. Gene ID is verified in Table S5. Samples are identified as C (control) or T (treated) followed by the time point and the day after harvested (DAH).

Some of these genes had their expression confirmed by qPCR (Figure 7) using the control versus 12 h ethylene treatment of RNA-seq experiment while also using a regular ripening curve (Figure S5). These results correspond to the observations in the RNA-seq and in the correlation analyses, in which papayas are indeed climacteric fruit with fast softening achieved exclusively by the massive action of the ethylene-induced set of PG [18,22,33].

The overview of all transcriptomics studies from papaya pulp (studies PRJNA352643, PRJNA449965, and PRJNA381300 https://trace.ncbi.nlm.nih.gov/Traces/sra/ accessed on 17 February 2020 [23,35,44]) including a meta-analyses with our results (PRJNA528193) with several statistical adjustments [27,29,46] generated a PCA plot to visualize the changes in all stages of data adjustment (Figure S6). Regarding gene expression profile of all transcriptomic data of papaya pulp, the division of clustered dendrogram in two subgroups (immature and ripe fruits) corroborates our data (Figure S7). Differentially expressed genes were identified between ripe and immature groups, and the expression comparison is shown for the main genes in Figure 4 and Figure 5 in the last column of the heat maps, corroborating our data.

The plant hormone ethylene is the main factor responsible for the fruit ripening phenomenon [2]. Transcriptomic data reveals that climacteric fruit distributes higher numbers of ethylene receptor genes; however, non-climacteric fruit expresses these genes at the onset of ripening. Therefore, it is reasonable to establish that this classification must be made upon fruit’s sensitivity to ethylene [47]. Ethylene is a major regulatory factor in climacteric fruit, not only in the ripening process but also in growth and development; it triggers a cascade of metabolic events that eventually result in cell wall disassembly [7].

Many cell wall modifying enzymes in fleshy fruit are ethylene-responsive [34]. The most explored pectin-modifying enzymes are PG, PL, and PME, although their individual actions are not enough to deeply modify cell wall structure [22]. Earlier studies using tomatoes have demonstrated that PG activities are three times greater in ripe fruit than in unripe fruit, following a similar pattern of ethylene production [48]. Another research study with mangoes revealed that PL and PG are active during ripening and remained with significative expression after the climacteric peak, but their expression was completely abolished after 1-MCP treatment [49,50]. Controversially, cell-wall-related genes from strawberry (a non-climacteric fruit) had a considerable increase in expression after 1-MCP treatment (PME, β-xylosidase, endoglucanase, xyloglucan endotransglycosidase/hydrolase, arabinofuranosidase, and cellulase genes), while they were downregulated after ethylene treatment [51]. In fact, non-climacteric fruit exhibited a relevant activity of PL together with PG, although the optimum pH of PL is significantly higher than that of PG.

**Figure 8:**
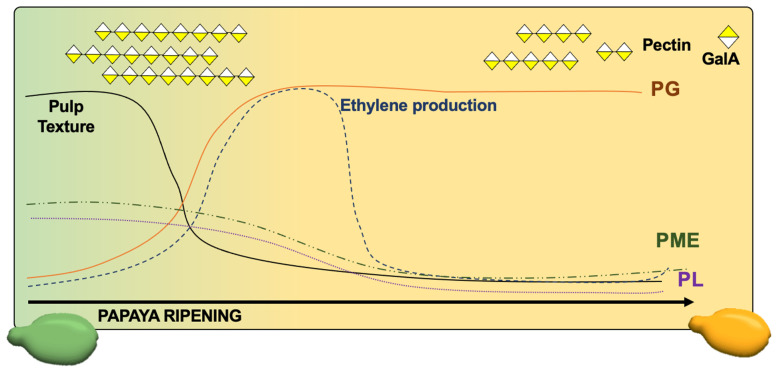
General representation of papaya fruit ripening and pulp softening. Papaya ripening is marked by a fast pulp softening due to a massive pectin mobilization driven by coordinated action of pectinases. While PMEs and PLs expressions decrease throughout ripening, PGs expression increases. GalA: galacturonic acid.

Papaya cell wall loosening in fleshy fruit and the consequent pulp softening are mainly achieved by the migration of pectin from insoluble to more soluble water fractions [22]. Moreover, papaya pulp softening is achieved by a coordinated action of cell wall related genes, that was proposed by our group just analyzing a very few sets of genes [33] that was corroborated by the transcriptomic data presented herein showing several genes. Climacteric fruit with fast pulp softening, such as papayas, present a basal expression of PME and decreased expression of PL but with a huge increment in endo-PG activity, leading to massive depolymerization and solubilization of pectins [22,33]. PG has a central role in depolymerizing the high molecular weight pectins from the chelate fractions to the water-soluble fractions, leading to a massive mobilization of these polysaccharides that culminates in pulp softening [22]. The present work reinforcers the ethylene action in triggering pectinases expressions, mainly PGs and galactosidases, demonstrating a tuned coordinate action of cell wall related gene expression to depolymerize high molecular weight pectins (Figure 8).

The mechanisms behind papaya pulp softening are different from other climacteric and non-climacteric fruit [44]. Slow pulp softening of non-climacteric fruit, such as strawberries, is marked by previous activity of PME and PL with a concomitant action of PG in ethylene-independent ways [52,53]. Slow pulp softening of climacteric fruit, such as some melons and peach varieties, demonstrates depolymerization in the later stage of pulp softening after PG-independent solubilization of pectins, probably through the cleavage of galactose residues from galactan side chains by galactosidases [54]. If peaches are hard-pulp varieties, there is only exo-PG activity with little depolymerization and solubilization of pectin [55]. Otherwise, if they are melting-pulp varieties, fast softening triggered by ethylene occurs through both exo- and endo-PG activities, leading to massive pectin depolymerization and solubilization, also with increased PME and galactosidases activities [55]. Apple is a climacteric hard-pulp fruit which pulp softening is also mainly due to the action of PG rather than PME, although it does not suffer significant depolymerization either of pectin or hemicellulose [54,56]. Climacteric fruits that have mid-fast pulp softening, such as tomatoes, had previous activities of PME and PL, just like strawberries, with pectin depolymerization at a mid-softening stage in a PG-dependent manner with galactanases activities appearing only after the onset of pulp softening [54,57]. All these comparisons show that pulp softening of fleshy fruit, especially papayas, is achieved by a coordinated action of several pectinases, but each fruit responds differently to ethylene stimuli leading to different grades of pulp texture.

## 4. Conclusions

This work describes a transcriptomic approach to study the ethylene-driven transcriptional expression in papayas highlighting the most significant changes and genes regulation. Differences between control and treated samples confirm the efficiency of exogenous ethylene treatment to induce ripening. Genes related to ethylene metabolism, carotenoids, plant cell wall modifications, response to several stimuli, and transcription activity have been identified and described. Co-expression correlation networks show the interconnected processes coordinated by many ethylene-dependent enzymes, especially the cell wall related ones. Papaya pulp softening is achieved by a coordinated action of several cell wall related genes, most of them belonging to the classification of pectinases. While PG and galactosidases expression increases during ripening, PME and PL decreases. The data provided in this work increases the knowledge of molecular mechanisms of ethylene’s role in the ripening process and enables further comprehensive studies to minimize papaya post-harvest losses. Further studies must be performed to completely elucidate the molecular and biochemical profiles of ripening-induced modifications of papayas for both economical and biotechnological purposes.

## Data Availability

The data presented in this study are openly available in GEO at reference number GSE128577. Publicly available datasets were analyzed in this study. This data can be found here: PRJNA352643, PRJNA449965, and PRJNA381300.

## Supplementary Materials

The following are available online at https://www.mdpi.com/article/10.3390/cells10092339/s1. Supplementary Material 1: Figure S1: Sampling scheme; Figure S2: Biological triplicate ripening parameters; Figure S3: GO terms distribution per category; Figure S4: PCA analysis of all samples DEGs; Figure S5: Ripening parameters of samples used to validation with qPCR; Figure S6: PCA plots of the steps for normalization of data; Figure S7: Dendrogram of meta-analysis data; Table S1: Nucleotide sequences used in qPCR; Table S2: GenBank Annotations; Table S3: Calibration curves for relative gene expression; Table S4: Differentially Expressed Genes (DEG) related to ethylene; Table S5: Differentially Expressed Genes (DEG) related to cell wall metabolism; Supplementary File 2: total DEGs between sample comparisons; Supplementary File 3: Number and PGs interactions with other cell wall genes.
