# Supplementary material for: Systems Biology Applied to the Study of Papaya Fruit Ripening: The Influence of Ethylene on Pulp Softening
Source: Cells. 2021 Sep 7;10(9):2339. doi: 10.3390/cells10092339 (PMC8467500; doi:10.3390/cells10092339)
Supplement: Supplementary file 1 [file cells-10-02339-s001.zip › Supplementary_Materials.pdf]

cells

MDPI

Article

Systems Biology Applied to the Study of Papaya Fruit Ripening: The Influence of Ethylene on Pulp Softening

Caroline Giacomelli Soares [1,2], Samira Bernardino Ramos do Prado [1,2], Sónia C.S. Andrade [3] and João Paulo Fabi [1,2,4,*]

[1] Department of Food Science and Experimental Nutrition, School of Pharmaceutical Sciences, University of São Paulo, São Paulo 05508-000, Brazil; cagias@usp.br (C.G.S.); samira.prado@oru.se (S.B.R.d.P.)
[2] Food Research Center (FoRC), CEPID-FAPESP (Research, Innovation and Dissemination Centers, São Paulo Research Foundation), São Paulo 05508-080, Brazil
[3] Departamento de Genética e Biologia Evolutiva, Instituto de Biociências, Universidade São Paulo, São Paulo 05508-060, Brazil; soniacsandrade@ib.usp.br
[4] Food and Nutrition Research Center (NAPAN), University of São Paulo, São Paulo 05508-060, Brazil
* Correspondence: author: jpfabi@usp.br; Tel.: +55-11-30913647

Abstract: Papaya is a fleshy fruit that undergoes fast ethylene-induced modifications. The fruit becomes edible, but the fast pulp softening is the main factor that limits the post-harvest period. Papaya fast pulp softening occurs due to cell wall disassembling coordinated by ethylene triggering that massively expresses pectinases. In this work, RNA-seq analysis of ethylene-treated and non-treated papayas enabled a wide transcriptome overview that indicated the role of ethylene during ripening at the gene expression level. Several families of transcription factors (AP2/ERF, NAC and MADS-box) were differentially expressed. ACO, ACS, and SAM-Mtase genes were up-regulated, indicating a high rate of ethylene biosynthesis after ethylene treatment. The correlation among gene expression and physiological data demonstrated ethylene treatment can indeed simulate ripening, and regulation of changes in fruit color, aroma and flavor could be attributed to the coordinated expression of several related genes. Especially about pulp firmness, the identification of 157 expressed genes related to cell wall metabolism demonstrated that unlike other climacteric and non-climacteric fruits, papaya does not need a previous action of pectate lyases and polygalacturonases are the main set of enzymes responsible for plant cell wall disassembling. The mechanism is systematically different for other commercially important fruits, such as strawberry, tomato, kiwifruit, and apple. The observed behavior of this new transcriptomic data is corroborated by the meta-analysis of several papaya transcriptomes, which confirmed ethylene triggering is the main event that elicits fast pulp softening in papayas.

Keywords: systems biology; papaya pulp softening; ethylene; post-harvest ripening-related modifications; pectinases; cell wall; climacteric fruit; non-climacteric fruit.

Supplementary Material

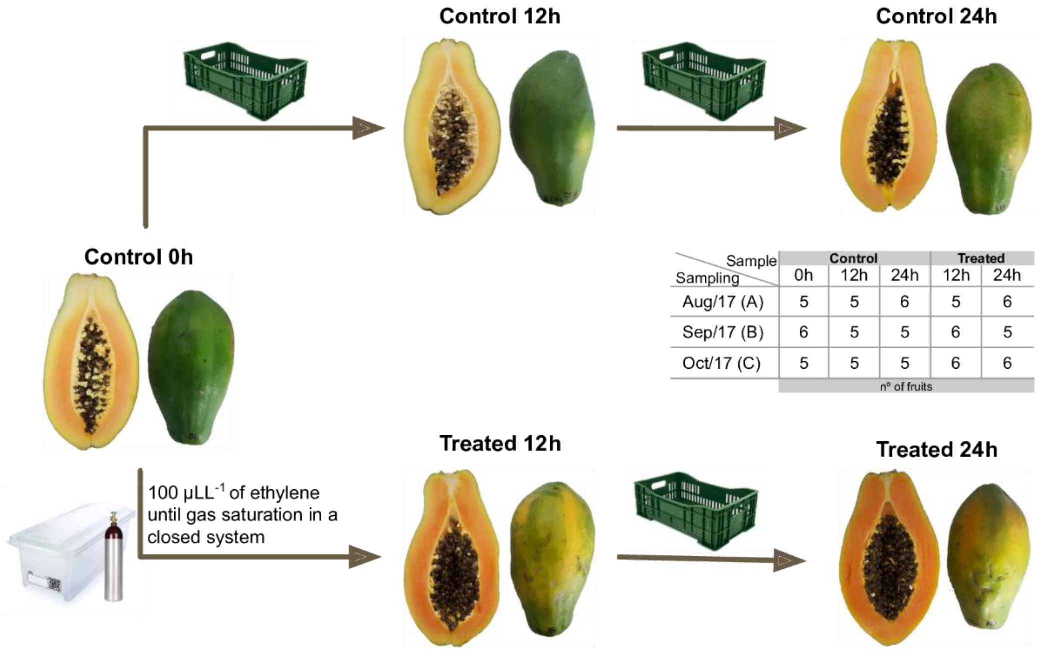

Figure S1. Sampling scheme. Scheme for dividing groups and sampling points. Each group consists of at least five fruits. This process was repeated three times, resulting in a biological triplicate.

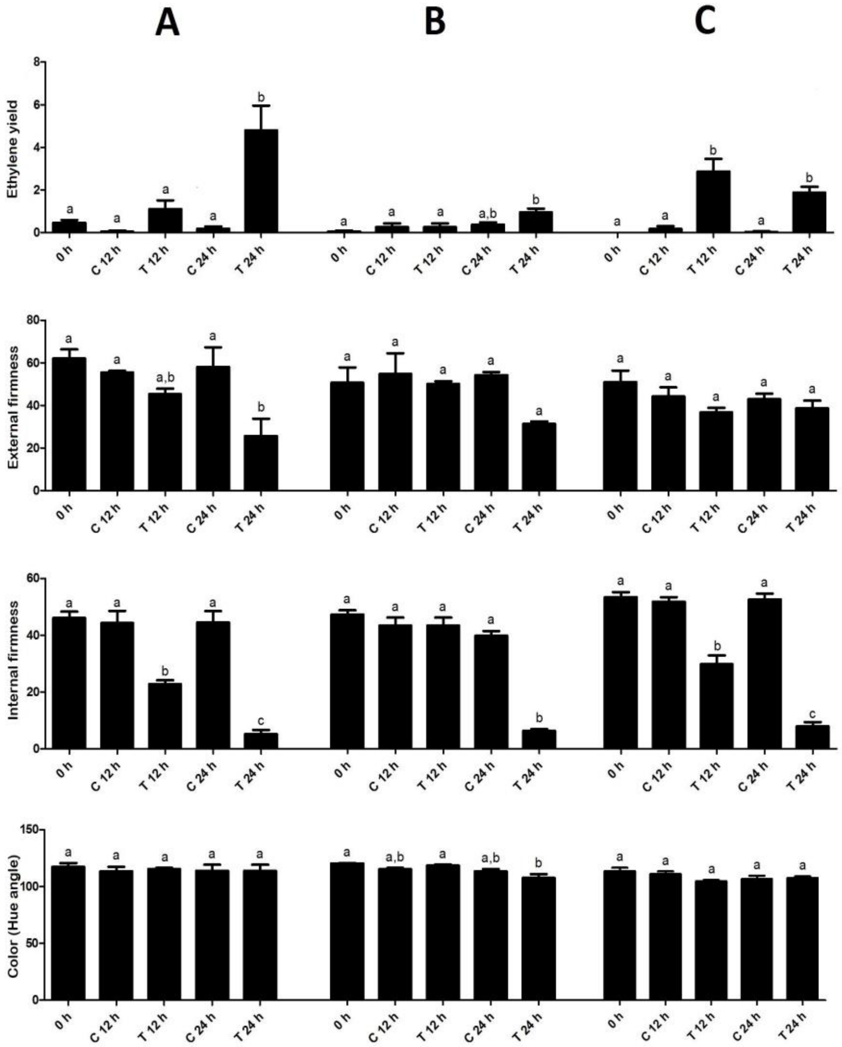

Figure S2. Ripening parameters measured for papaya treatment for all samplings. Ripening parameters: ethylene, (μL.Kg^{-1}.h^{-1}), external firmness (N), internal firmness (N), and pulp color (hue angle). Five to six papayas were used for each time point (0 h, 12 h, and 24 h) for each biological replicate (**A**, **B**, and **C**). Samples are identified as C (control) or T (treated) followed by the time point.

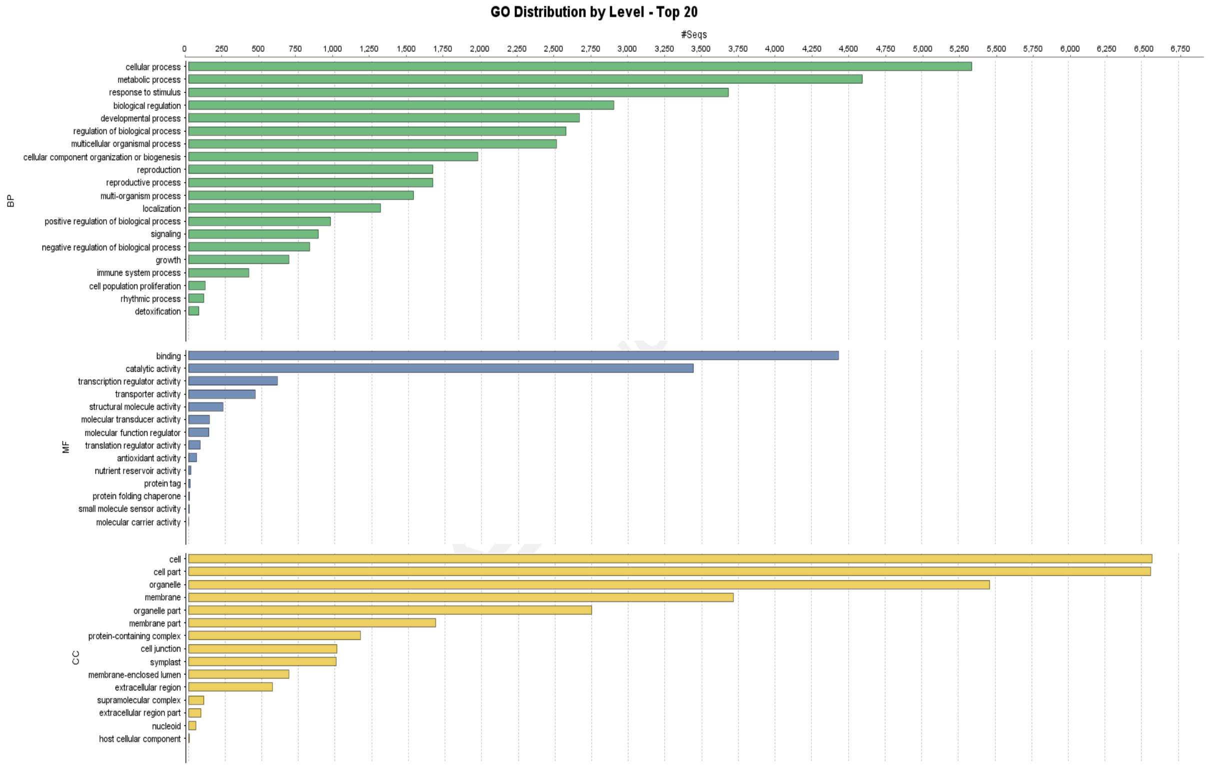

Figure S3. GO terms distribution per category. For each category, the 20 GO terms with the highest number of genes are represented. BP: Biological Process, MF: Molecular Function, CC: Cellular Component.

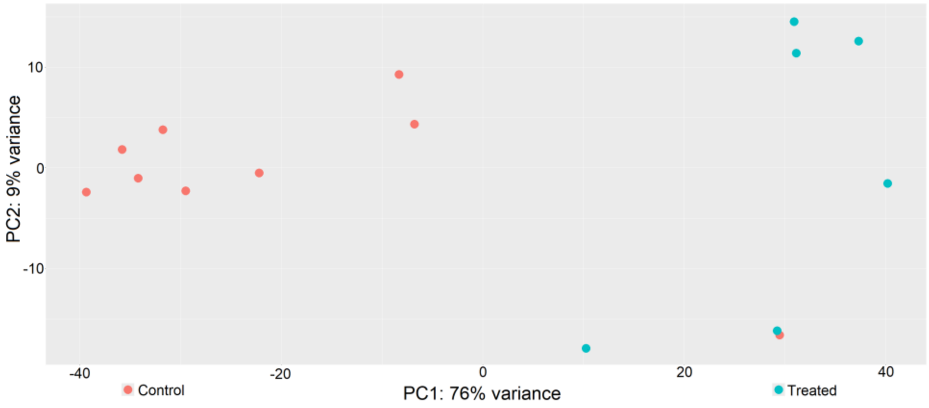

Figure S4. Principal Component Analysis of differentially expressed genes in papaya pulp. Total number of differentially expressed genes with p-adj ≤ 0.05 and $\log_2 FC > |1.5|$ between pairwise comparisons were compared. Samples are identified as red dots for control and blue dots for treated.

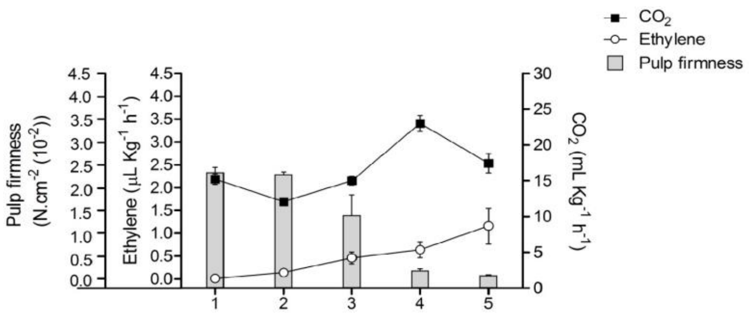

Figure S5. Ripening parameters of papaya samples used in qPCR experiments.

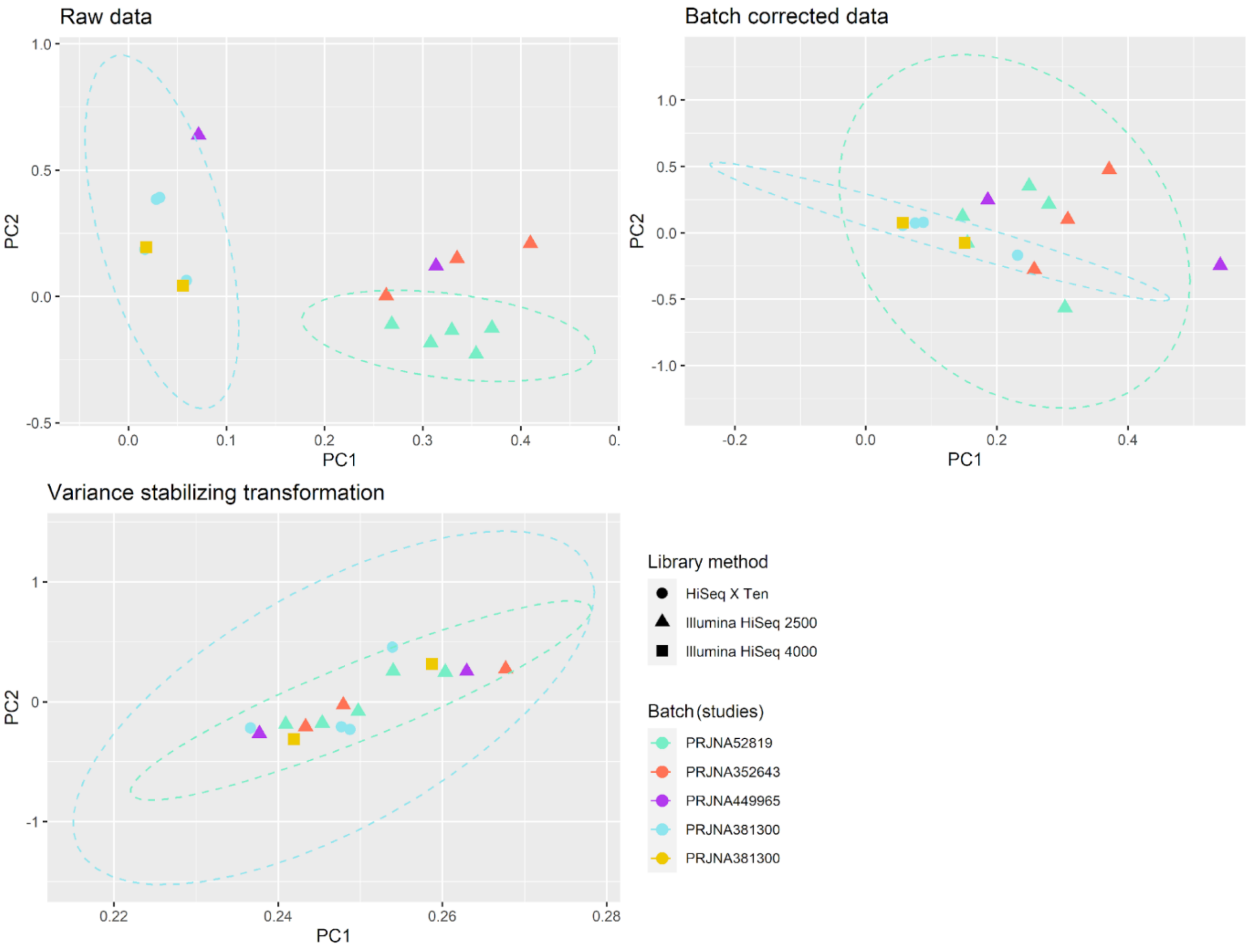

Figure S6. PCA plots of the steps to normalize the data. It was considered the differences between the platforms of sequencing, as well as the studies (batch).

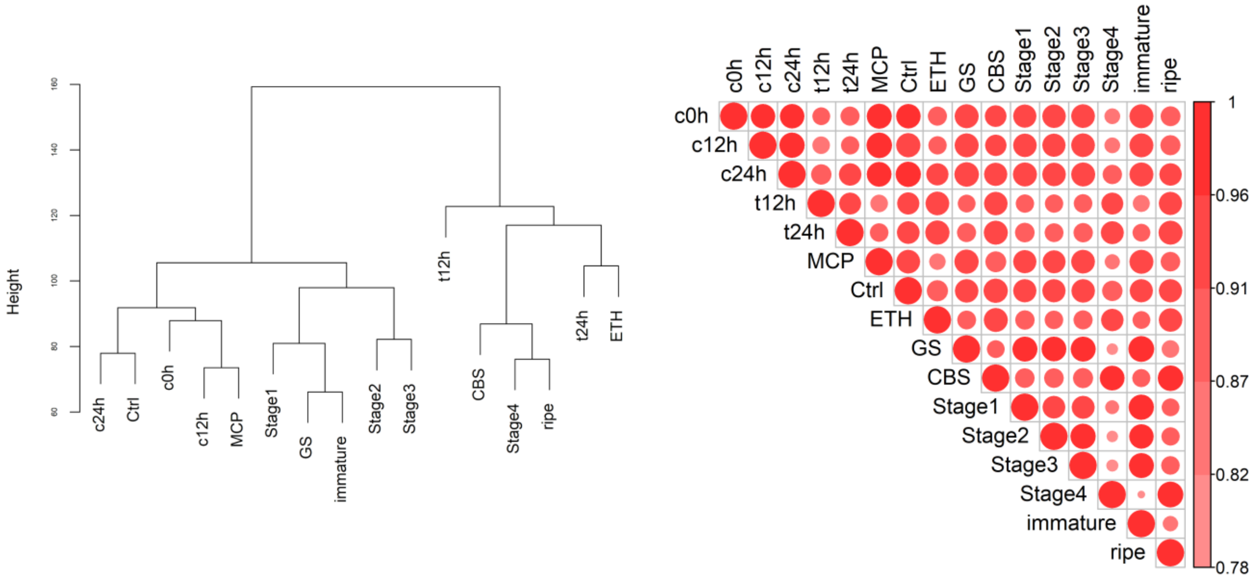

(**a**) (**b**)

Figure S7. Cluster dendrogram and Pearson correlation of papaya transcriptomes data from data integration. Sample tags are depicted in Table 1. Circle size and color represent the correlation between samples.

Table S1. Nucleotide sequences used in qPCR.

Gene	Sequence (5′→3′)
PG1	TGG TGG TGC GTA TAG ATG GA<br>ACA AAA CCC AGT ACC CAC CA
PG2	TCC TGA AGC TCA CCC TTC AT<br>CCT CAA TGC CTT TGA AGC TC
PG3	TTG GAG GGC AGC TTG TTT AG<br>CAC CCA AGC CTT TAT TGT TCC
PL1	TTC CCT GTG GGC TTA CAA TC<br>AGG GAG GTC TGC CAT TAC AT
PL2	CAT GTT CTT GTC CTG CGT GT<br>CCA TCC ACG GCT CTA ATT TC
AGAL1	GTA TAG GCG GAA GGT GGA T<br>AAG GCC ACC TCT CTG GAT
AGAL3	TGT CCG TTG ATC CTT CAG TG<br>AGT CCG GAA GAA TGC TGA TG
BGAL1	GTG CTT GCA ACT ATG CTG GA<br>ATA GGT TCG CAG TTG GGT TG
BGAL3	CCA AAG TGG GGA CAT TTG AG<br>ACA CCC AGA CTT CGA CTT G
PME1	TAT CTT GGT AGG CCC TGG A<br>AGG CCA GTG TTT CGG TAC T
PME2	GTG GTT TGT TCC TCA GCA CA<br>TGG AAC GTA ACT GCA AGT GG
PME3	GCA AGC TTT AGG GGT GTT GA<br>AGG CCT GCA GAG CTT ATT GA
ACT	CGT GAC CTT ACT GAT CAC TTG<br>GTC AAG GGC AAT GTA AGA CAG
EF1	GTT AAG AAC GTT GCC GTG AAG<br>ATG TGA AGT TGG CTG CTT CCT
UBQ	ACT CAC CGG CAA GAC CAT<br>GTG GAG AGT CGA TTC CTT TTG

Table S2. GenBank Annotations.

Gene	Accession number
PG1	FJ007644
PG2	GQ479791
PG3	GQ479794
PL1	DQ660903
PL2	ABIM01001816
AGAL1	ABIM01008846
AGAL3	ABIM01016598
BGAL1	AF064786
BGAL3	ABIM01026480
PME1	GR486204
PME2	ABIM01018702
PME3	ABIM01014785

Table S3. Calibration curves for relative gene expression.

Gene	Efficiency (10-slope)	y = ax+b	R^2
PG1	1.96	-3.4408x + 22.295	1
PG2	1.94	-3.4841x + 27.584	0.99
PG3	1.93	-3.4997x + 28.439	1
PL1	1.98	-3.3722x + 23.508	1
PL2	1.82	-3.8546x + 35.543	0.99
AGAL1	2.2	-2.9239x + 25.249	0.99
AGAL3	2.17	-2.9673x + 25.612	0.97
BGAL1	2.05	-3.2145x + 24.034	0.99
BGAL3	1.93	-3.4933x + 25.775	1
PME1	1.95	-3.4408x + 22.295	1
PME2	1.94	-3.4841x + 27.584	0.99
PME3	1.93	-3.4997x + 28.439	1
ARF	2	-3.3321x + 27.523	0.99
XYL	1.94	-3.4742x + 23.389	1
CELL	1.86	-3.7239x + 24.695	0.99
XTH	1.74	-4.1503x + 28.827	0.99
ACT	2.03	-3.2446x + 21.922	0.99
EF1	1.92	-3.5886x + 25.540	0.98
UBQ	1.89	-3.4841x + 22.049	0.99

Table S4. Differentially expressed genes related to ethylene.

Gene	Gene ID (NCBI)	Putative Function	Coding Enzyme
gene11206	LOC110815924	1-aminocyclopropane-1-carboxylate oxidase homolog 1-like	ACO
gene11209	LOC110815808	1-aminocyclopropane-1-carboxylate oxidase homolog 1-like	ACO
gene18244	LOC110823870	1-aminocyclopropane-1-carboxylate oxidase homolog 4-like	ACO
gene19881	LOC110825516	1-aminocyclopropane-1-carboxylate oxidase homolog 1-like	ACO
gene19882	LOC110825518	1-aminocyclopropane-1-carboxylate oxidase homolog 1-like	ACO
gene3976	LOC110807747	1-aminocyclopropane-1-carboxylate oxidase homolog 1-like isoform X1	ACO
gene4262	LOC110808078	1-aminocyclopropane-1-carboxylate oxidase	ACO
gene4917	LOC110808805	1-aminocyclopropane-1-carboxylate oxidase	ACO
gene6521	LOC110810572	1-aminocyclopropane-1-carboxylate oxidase homolog 4-like	ACO
gene9174	LOC110813532	1-aminocyclopropane-1-carboxylate oxidase 5	ACO
gene12660	LOC110817493	probable aminotransferase ACS10	ACS
gene1560	LOC110819154	1-aminocyclopropane-1-carboxylate synthase-like	ACS
gene17898	LOC110823221	probable aminotransferase ACS12 isoform X2	ACS
gene821	LOC110811354	1-aminocyclopropane-1-carboxylate synthase 1-like	ACS
gene6573	LOC110810650	protein ethylene insensitive 3-like	EIN
gene7653	LOC110811898	ethylene insensitive 3a	EIN
gene9970	LOC110814495	ethylene insensitive 3-like 1 protein	EIN
gene11078	LOC110815652	AP2/ERF and B3 domain-containing transcription factor At1g50680-like	ERF/AP2
gene1120	LOC110814499	ethylene-responsive transcription factor RAP2-13-like	ERF/AP2
gene11679	LOC110816409	ethylene-responsive transcription factor ERF113	ERF/AP2
gene11897	LOC110816728	ethylene-responsive transcription factor ERF014-like	ERF/AP2
gene11958	LOC110816696	ethylene-responsive transcription factor RAP2-4	ERF/AP2
gene12180	LOC110817045	ethylene-responsive transcription factor ERF054-like	ERF/AP2
gene13844	LOC110818891	ethylene-responsive transcription factor RAP2-1-like	ERF/AP2
gene15594	LOC110820757	transcription termination factor MTERF9	ERF/AP2
gene16077	LOC110821290	transcription termination factor MTERF4	ERF/AP2
gene1813	LOC110822141	ethylene-responsive transcription factor RAP2-12-like	ERF/AP2
gene18563	LOC110824230	ethylene-responsive transcription factor ERF119	ERF/AP2
gene19906	LOC110825544	ethylene-responsive transcription factor ERF036-like	ERF/AP2
gene2448	LOC110826171	ethylene-responsive transcription factor ERF016-like	ERF/AP2
gene2737	LOC110806359	AP2-like ethylene-responsive transcription factor At2g41710 isoform X1	ERF/AP2
gene3058	LOC110806714	AP2-like ethylene-responsive transcription factor At1g16060	ERF/AP2
gene426	LOC110807473	ethylene-responsive transcription factor RAP2-3-like	ERF/AP2
gene4688	LOC110808549	ethylene-responsive transcription factor RAP2-7	ERF/AP2
gene7173	LOC110811357	ethylene-responsive transcription factor ERF015	ERF/AP2
gene7636	LOC110811803	AP2-like ethylene-responsive transcription factor PLT2 isoform X2	ERF/AP2
gene8556	LOC110812914	ethylene-responsive transcription factor ABR1-like	ERF/AP2
gene9224	LOC110813758	ethylene-responsive transcription factor ERF109-like	ERF/AP2
gene10466	LOC110815052	agamous-like MADS-box protein AGL1 isoform X1	MADS
gene1060	LOC110813824	agamous-like MADS-box protein AGL62	MADS
gene11347	LOC110815973	agamous-like MADS-box protein AGL62	MADS
gene1150	LOC110814794	MADS-box transcription factor 27-like	MADS
gene12638	LOC110817459	agamous-like MADS-box protein AGL19	MADS
gene13711	LOC110818772	MADS-box protein AGL24-like isoform X1	MADS
gene1518	LOC110818645	MADS-box protein CMB1	MADS
gene17642	LOC110823018	MADS-box protein AGL42	MADS
gene1831	LOC110822337	agamous-like MADS-box protein AGL11	MADS
gene18455	LOC110824197	agamous-like MADS-box protein AGL30 isoform X2	MADS
gene19366	LOC110824954	agamous-like MADS-box protein AGL18	MADS
gene3579	LOC110807301	MADS-box transcription factor 23-like isoform X3	MADS
gene5301	LOC110809260	agamous-like MADS-box protein AGL30	MADS
gene8906	LOC110813260	agamous-like MADS-box protein AGL104	MADS
gene9820	LOC110814435	MADS-box protein SVP	MADS
gene10128	LOC110814672	NAC domain-containing protein 78-like	NAC
gene11472	LOC110816152	NAC domain-containing protein 82-like	NAC
gene11492	LOC110816201	NAC domain-containing protein 104-like	NAC
gene12931	LOC110817727	NAC transcription factor 25	NAC
gene13848	LOC110818781	NAC domain-containing protein 72-like	NAC
gene1443	LOC110817864	NAC domain-containing protein 53-like	NAC
gene15050	LOC110820288	NAC domain-containing protein 100-like	NAC
gene1617	LOC110819852	NAC domain-containing protein 79-like	NAC
gene17606	LOC110822981	NAC domain-containing protein 83	NAC

gene17830	LOC110823491	NAC domain-containing protein 62-like	NAC
gene18457	LOC110824264	NAC transcription factor 29-like	NAC
gene2236	LOC110825934	NAC domain-containing protein 35	NAC
gene3001	LOC110806658	NAC domain-containing protein 62-like	NAC
gene3162	LOC110806832	NAC domain-containing protein 90	NAC
gene375	LOC110807011	NAC domain-containing protein 10	NAC
gene3886	LOC110807683	NAC domain-containing protein 21/22-like isoform X2	NAC
gene5914	LOC110809964	NAC domain-containing protein 102-like	NAC
gene6043	LOC110810112	NAC domain-containing protein 100	NAC
gene6386	LOC110810455	NAC domain-containing protein 41-like isoform X1	NAC
gene6701	LOC110810834	NAC domain-containing protein 83	NAC
gene6988	LOC110811156	NAC domain-containing protein 30-like	NAC
gene7210	LOC110811410	NAC domain-containing protein 71-like	NAC
gene8687	LOC110812963	NAC domain-containing protein 83	NAC
gene9094	LOC110813524	NAC domain-containing protein 30-like	NAC
gene9148	LOC110813582	NAC domain-containing protein 2-like	NAC
gene10013	LOC110814610	S-adenosylmethionine decarboxylase proenzyme	SAM
gene11023	LOC110815710	uncharacterized methyltransferase At2g41040	SAM
gene11505	LOC110816192	putative methyltransferase DDB_G0268948	SAM
gene11924	LOC110816733	S-adenosyl-L-methionine-dependent tRNA 4-demethylwyosine synthase	SAM
gene11925	LOC110816673	probable methyltransferase PMT28	SAM
gene11991	LOC110816762	probable pectin methyltransferase QUA2	SAM
gene12458	LOC110817288	uncharacterized protein LOC110817288	SAM
gene12468	LOC110817300	uncharacterized protein LOC110817300 isoform X1	SAM
gene13105	LOC110818250	uncharacterized protein LOC110818250	SAM
gene13140	LOC110818142	probable methyltransferase PMT24	SAM
gene13163	LOC110818280	probable methyltransferase PMT27	SAM
gene13799	LOC110818807	uncharacterized protein At2g23090-like	SAM
gene14221	LOC110819234	probable methyltransferase PMT22	SAM
gene1430	LOC110817772	S-adenosylmethionine decarboxylase proenzyme-like	SAM
gene14343	LOC110819192	probable methyltransferase PMT11	SAM
gene14524	LOC110819466	probable methyltransferase PMT13	SAM
gene14528	LOC110819609	probable methyltransferase PMT15	SAM
gene14638	LOC110819718	uncharacterized protein LOC110819718	SAM
gene15451	LOC110820875	methyltransferase-like protein 5 isoform X2	SAM
gene15803	LOC110821026	uncharacterized protein LOC110821026	SAM
gene1588	LOC110819478	probable methyltransferase PMT3	SAM
gene16266	LOC110821237	probable methyltransferase PMT27	SAM
gene16732	LOC110821949	uncharacterized protein LOC110821949	SAM
gene16996	LOC110822495	probable methyltransferase PMT27	SAM
gene17396	LOC110822617	putative methyltransferase DDB_G0268948 isoform X1	SAM
gene17397	LOC110822698	putative methyltransferase DDB_G0268948	SAM
gene17551	LOC110823023	uncharacterized protein LOC110823023	SAM
gene1771	LOC110821716	probable methyltransferase PMT20	SAM
gene19264	LOC110824843	probable S-adenosylmethionine-dependent methyltransferase At5g38780	SAM
gene19467	LOC110825064	probable methyltransferase PMT21	SAM
gene2071	LOC110825210	uncharacterized protein LOC110825210	SAM
gene2090	LOC110825401	probable methyltransferase PMT18	SAM
gene250	LOC110825890	probable methyltransferase PMT26	SAM
gene3697	LOC110807416	uncharacterized protein LOC110807416 isoform X2	SAM
gene3709	LOC110807474	probable S-adenosylmethionine-dependent methyltransferase At5g37990	SAM
gene3807	LOC110807564	uncharacterized protein LOC110807564	SAM
gene4479	LOC110808295	S-adenosylmethionine synthase 2	SAM
gene4610	LOC110808470	uncharacterized protein LOC110808470	SAM
gene5085	LOC110808988	probable methyltransferase PMT9	SAM
gene5189	LOC110809125	probable methyltransferase PMT2	SAM
gene5374	LOC110809320	methyltransferase-like protein 13	SAM
gene6027	LOC110810110	probable methyltransferase PMT2 isoform X1	SAM
gene6206	LOC110810353	probable methyltransferase PMT15 isoform X1	SAM
gene6624	LOC110810745	S-adenosylmethionine synthase 3	SAM
gene7179	LOC110811374	(S)-coclaurine N-methyltransferase-like	SAM
gene7691	LOC110811946	methyltransferase-like protein 13	SAM
gene8021	LOC110812319	uncharacterized protein LOC110812319	SAM
gene8617	LOC110813022	S-adenosylmethionine decarboxylase proenzyme-like	SAM
gene8823	LOC110813193	S-adenosylmethionine synthase	SAM

gene9683	LOC110814181	uncharacterized protein LOC110814181 isoform X2	SAM

Table S5. Differentially expressed genes related to cell wall metabolism during papaya ripening.

Gene	Gene ID (NCBI)	Putative Function	Coding Enzyme
gene1360	LOC110817097	alpha-galactosidase 1-like	A-GAL
gene14164	LOC110819226	alpha-galactosidase 3	A-GAL
gene3830	LOC110807600	mannosyl-oligosaccharide 1,2-alpha-mannosidase MNS3, partial	A-MANN
gene6084	LOC110810147	alpha-mannosidase I MNS5 isoform X1	A-MANN
gene6889	LOC110811093	mannosyl-oligosaccharide 1,2-alpha-mannosidase MNS1-like isoform X1	A-MANN
gene879	LOC110811995	mannosyl-oligosaccharide 1,2-alpha-mannosidase MNS1-like	A-MANN
gene8799	LOC110813200	mannosyl-oligosaccharide 1,2-alpha-mannosidase MNS2-like, partial	A-MANN
gene9560	LOC110814068	alpha-mannosidase I MNS4	A-MANN
gene1032	LOC110813559	lysine-rich arabinogalactan protein 18	AGP
gene10377	LOC110814932	arabinogalactan peptide 13-like	AGP
gene11392	LOC110816092	classical arabinogalactan protein 9-like	AGP
gene11393	LOC110816167	classical arabinogalactan protein 1	AGP
gene12026	LOC110816610	classical arabinogalactan protein 10	AGP
gene12118	LOC110816923	fasciclin-like arabinogalactan protein 21	AGP
gene12343	LOC110817139	fasciclin-like arabinogalactan protein 17	AGP
gene12891	LOC110817819	non-classical arabinogalactan protein 31-like	AGP
gene14281	LOC110819182	Fasciclin-like arabinogalactan family protein	AGP
gene14835	LOC110819981	classical arabinogalactan protein 27	AGP
gene15557	LOC110820823	fasciclin-like arabinogalactan protein 1	AGP
gene17706	LOC110823186	arabinogalactan peptide 22-like	AGP
gene18615	LOC110824087	lysine-rich arabinogalactan protein 19	AGP
gene2775	LOC110806382	fasciclin-like arabinogalactan protein 17	AGP
gene2830	LOC110806455	classical arabinogalactan protein 7-like	AGP
gene3877	LOC110807696	fasciclin-like arabinogalactan protein 13	AGP
gene4805	LOC110808736	fasciclin-like arabinogalactan protein 2	AGP
gene5562	LOC110809511	arabinogalactan peptide 13-like	AGP
gene803	LOC110811190	fasciclin-like arabinogalactan protein 4 isoform X1	AGP
gene12218	LOC110816900	alpha-L-arabinofuranosidase 1-like	ARA
gene12413	LOC110817099	alpha-L-arabinofuranosidase 1-like isoform X2	ARA
gene11696	LOC110816435	beta-galactosidase 10	B-GAL
gene11704	LOC110816515	beta-galactosidase 6 isoform X2	B-GAL
gene12775	LOC110817680	beta-galactosidase 15 isoform X1	B-GAL
gene12802	LOC110817677	beta-galactosidase 15 isoform X1	B-GAL
gene13406	LOC110818407	beta-galactosidase 1 isoform X1	B-GAL
gene1695	LOC110820693	uncharacterized protein LOC110820693	B-GAL
gene16987	LOC110822129	beta-galactosidase 3	B-GAL
gene2838	LOC110806458	beta-galactosidase 8	B-GAL
gene6671	LOC110810818	beta-galactosidase 5	B-GAL
gene7019	LOC110811182	beta-galactosidase-like	B-GAL
gene7693	LOC110811947	beta-galactosidase 9-like	B-GAL
gene9153	LOC110813600	beta-galactosidase 16 isoform X1	B-GAL
gene9155	LOC110813537	beta-galactosidase 16-like isoform X1	B-GAL
gene10728	LOC110815331	mannosylglycoprotein endo-beta-mannosidase	B-MANN
gene12216	LOC110816958	mannan endo-1,4-beta-mannosidase 2-like	B-MANN
gene16952	LOC110822316	mannan endo-1,4-beta-mannosidase 7	B-MANN
gene7425	LOC110811647	mannan endo-1,4-beta-mannosidase 6	B-MANN
gene940	LOC110812631	mannan endo-1,4-beta-mannosidase 1, partial	B-MANN
gene9675	LOC110814153	mannan endo-1,4-beta-mannosidase 1, partial	B-MANN
gene10588	LOC110815157	endoglucanase 25-like	CELL-ase
gene12309	LOC110817105	Cellulase (glycosyl hydrolase family 5) protein	CELL-ase
gene12310	LOC110817068	Cellulase (glycosyl hydrolase family 5) protein	CELL-ase
gene12311	LOC110817069	Cellulase (glycosyl hydrolase family 5) protein	CELL-ase
gene13783	LOC110818785	endoglucanase 3	CELL-ase
gene14581	LOC110819632	endoglucanase 25-like	CELL-ase
gene17040	LOC110822284	endoglucanase 24-like	CELL-ase
gene18257	LOC110823831	endoglucanase 8	CELL-ase
gene20068	LOC110825722	endoglucanase 6-like	CELL-ase
gene4328	LOC110808110	endoglucanase 25-like	CELL-ase
gene10632	LOC110815166	cellulose synthase A catalytic subunit 2 [UDP-forming]-like	CELL-syn
gene11115	LOC110815766	cellulose synthase A catalytic subunit 7 [UDP-forming]-like	CELL-syn
gene11930	LOC110816665	probable cellulose synthase A catalytic subunit 5 [UDP-forming] isoform X2	CELL-syn
gene13050	LOC110817890	cellulose synthase-like protein E6	CELL-syn

gene13051	LOC110817796	cellulose synthase-like protein E6 isoform X1	CELL-syn
gene147	LOC110817465	cellulose synthase A catalytic subunit 6 [UDP-forming]-like isoform X2	CELL-syn
gene14914	LOC110820068	cellulose synthase-like protein G3 isoform X1	CELL-syn
gene16477	LOC110821594	cellulose synthase, putative (DUF1644)	CELL-syn
gene19062	LOC110824624	cellulose synthase-like D3	CELL-syn
gene19957	LOC110825603	cellulose synthase A catalytic subunit 3 [UDP-forming]-like	CELL-syn
gene5787	LOC110809801	cellulose synthase A catalytic subunit 1 [UDP-forming]	CELL-syn
gene8551	LOC110812917	cellulose synthase A catalytic subunit 2 [UDP-forming]	CELL-syn
gene957	LOC110812813	cellulose synthase-like protein D2, partial	CELL-syn
gene10529	LOC110815026	exopolygalacturonase-like	exo-PG
gene10338	LOC110814908	expansin-A15-like	EXP
gene13333	LOC110818133	expansin-A11	EXP
gene14044	LOC110818908	expansin-like A2	EXP
gene14153	LOC110819370	expansin-A8	EXP
gene18313	LOC110823677	expansin-A8-like, partial	EXP
gene18763	LOC110824164	expansin-A4	EXP
gene19599	LOC110825207	expansin-like B1	EXP
gene2480	LOC110826208	expansin-A6-like	EXP
gene3555	LOC110807298	expansin-A13	EXP
gene10889	LOC110815466	pectin acetylesterase 12-like isoform X1	PAE
gene1193	LOC110815318	pectin acetylesterase 8 isoform X1	PAE
gene4550	LOC110808409	pectin acetylesterase 7-like	PAE
gene9661	LOC110814143	pectin acetylesterase 6-like isoform X1	PAE
gene12584	LOC110817416	polygalacturonase QRT3-like isoform X1	PG
gene12777	LOC110817723	probable polygalacturonase	PG
gene15943	LOC110821202	probable polygalacturonase At1g80170 isoform X2	PG
gene16066	LOC110821401	probable polygalacturonase	PG
gene1797	LOC110821988	probable polygalacturonase	PG
gene2536	LOC110826274	polygalacturonase-like	PG
gene3486	LOC110807205	probable polygalacturonase	PG
gene5336	LOC110809302	polygalacturonase QRT2-like	PG
gene7049	LOC110811267	polygalacturonase-like isoform X1	PG
gene9058	LOC110813488	polygalacturonase-like	PG
gene9059	LOC110813489	polygalacturonase-like	PG
gene9649	LOC110814146	probable polygalacturonase	PG
gene14785	LOC110819881	polygalacturonase inhibitor protein, partial	PGI
gene19582	LOC110825189	polygalacturonase inhibitor (DUF639)	PGI
gene19834	LOC110825467	polygalacturonase inhibitor (DUF639)	PGI
gene38	LOC110807182	polygalacturonase inhibitor (DUF639)	PGI
gene10120	LOC110814542	probable pectate lyase 13	PL
gene14718	LOC110819800	pectate lyase-like	PL
gene16482	LOC110821787	probable pectate lyase 8 isoform X2	PL
gene171	LOC110819499	probable pectate lyase 4, partial	PL
gene17454	LOC110822705	probable pectate lyase 3	PL
gene17545	LOC110823044	probable pectate lyase 5	PL
gene4246	LOC110808054	putative pectate lyase 2, partial	PL
gene9513	LOC110813995	probable pectate lyase 1	PL
gene10769	LOC110815463	pectinesterase (Protein of unknown function, DUF538)	PME
gene10836	LOC110815417	probable pectinesterase 29	PME
gene13357	LOC110818178	putative pectinesterase 11	PME
gene13517	LOC110818404	pectinesterase 1	PME
gene14736	LOC110819677	pectinesterase 1	PME
gene15057	LOC110820172	pectinesterase 31	PME
gene16191	LOC110821415	pectinesterase (Protein of unknown function, DUF538)	PME
gene16192	LOC110821414	pectinesterase (Protein of unknown function, DUF538)	PME
gene17689	LOC110823327	putative pectinesterase 63	PME
gene3087	LOC110806763	pectinesterase	PME
gene4292	LOC110808164	pectinesterase-like	PME
gene4527	LOC110808384	probable pectinesterase 53	PME
gene10533	LOC110815072	probable pectinesterase/pectinesterase inhibitor 58	PMEI
gene10774	LOC110815485	pectinesterase inhibitor-like	PMEI
gene11454	LOC110816184	probable pectinesterase/pectinesterase inhibitor 51	PMEI
gene12359	LOC110817077	probable pectinesterase/pectinesterase inhibitor 46	PMEI
gene12707	LOC110817457	pectinesterase inhibitor 10	PMEI
gene12708	LOC110817609	pectinesterase inhibitor 9-like isoform X3	PMEI

gene13023	LOC110817778	pectinesterase inhibitor 5-like	PMEI
gene15998	LOC110821276	pectinesterase inhibitor-like	PMEI
gene17738	LOC110823232	probable pectinesterase/pectinesterase inhibitor 34	PMEI
gene17935	LOC110823499	pectinesterase inhibitor 4	PMEI
gene17936	LOC110823071	pectinesterase inhibitor 11	PMEI
gene17939	LOC110823299	probable pectinesterase/pectinesterase inhibitor 59	PMEI
gene4520	LOC110808377	probable pectinesterase/pectinesterase inhibitor 36	PMEI
gene4522	LOC110808380	pectinesterase inhibitor 9	PMEI
gene699	LOC110810171	probable pectinesterase/pectinesterase inhibitor 12	PMEI
gene19959	LOC110825605	probable rhamnogalacturonate lyase B, partial	rglB
gene19960	LOC110825606	probable rhamnogalacturonate lyase B, partial	rglB
gene3582	LOC110807303	probable rhamnogalacturonate lyase B isoform X1	rglB
gene12117	LOC110817030	xyloglucan endotransglucosylase/hydrolase protein 9	XTH
gene16436	LOC110821790	putative xyloglucan endotransglucosylase/hydrolase protein 1	XTH
gene18197	LOC110823748	probable xyloglucan endotransglucosylase/hydrolase protein 28	XTH
gene18427	LOC110824296	xyloglucan endotransglucosylase/hydrolase protein 31	XTH
gene2660	LOC110826398	probable xyloglucan endotransglucosylase/hydrolase protein 25	XTH
gene2663	LOC110826405	xyloglucan endotransglucosylase/hydrolase protein 24-like	XTH
gene2664	LOC110826410	probable xyloglucan endotransglucosylase/hydrolase protein 23	XTH
gene2950	LOC110806596	probable xyloglucan endotransglucosylase/hydrolase protein 30	XTH
gene3084	LOC110806756	probable xyloglucan endotransglucosylase/hydrolase protein 8	XTH
gene5145	LOC110809080	probable xyloglucan endotransglucosylase/hydrolase protein 6	XTH
gene6942	LOC110811082	probable xyloglucan endotransglucosylase/hydrolase protein 23	XTH
gene8626	LOC110813037	probable xyloglucan endotransglucosylase/hydrolase protein 5	XTH
gene12726	LOC110817606	probable xyloglucan galactosyltransferase GT19	XYG
gene17892	LOC110823425	xyloglucan galactosyltransferase XLT2	XYG
gene4133	LOC110807966	probable xyloglucan galactosyltransferase GT14	XYG
gene4134	LOC110807938	probable xyloglucan galactosyltransferase GT14	XYG
gene7317	LOC110811520	xyloglucan galactosyltransferase MUR3, partial	XYG
gene7908	LOC110812108	probable xyloglucan galactosyltransferase GT11	XYG
